# Antibody-dependent cellular phagocytosis in cancer immunotherapy: research and perspectives

**DOI:** 10.1186/s12967-026-08020-5

**Published:** 2026-03-19

**Authors:** Qihong Gu, Siqi Li, Shiqiang Hou, Jiahao Liu, Xinyang Zhang, Yan Xi, Hongzhao Chen, Songbai Liu, Minjie Chu, Minfeng Yang

**Affiliations:** 1 School of Public Health, Nantong University, Nantong, China; 2 Suzhou Key Laboratory of Medical Biotechnology, Suzhou Vocational Health College, Suzhou, China; 3 The Affiliated Chuzhou Hospital of Anhui Medical University, The First People’s Hospital of Chuzhou, Chuzhou, China; 4 Medical School of Nantong University, Nantong, China; 5 Department of Health Technology and Informatics, The Hong Kong Polytechnic University, Kowloon, Hong Kong SAR P.R. China

**Keywords:** Cancer immunotherapy, Antibody-dependent cell phagocytosis, Macrophages, Intravital imaging

## Abstract

**Background:**

Cancer immunotherapy has revolutionized the clinical management of cancer due to its promising survival benefits. Although FDA-approved cancer immunotherapy primarily leverages adaptive immunity for therapeutic efficacy, the critical role of innate immunity in tumor surveillance and eradication has been increasingly recognized. Recently, with the deepening of research in this field, macrophages have emerged as key effectors of the innate anti-tumor response, with antibody-dependent cellular phagocytosis (ADCP) becoming one of the primary mechanisms involved in cancer immunotherapy. As such, exploring ADCP-inducing monoclonal antibodies (mAbs) as forefront therapeutic regimens for patients with cancer is therefore highly sought after.

**Main body:**

Here, we broadly summarize recent translational advances in harnessing macrophage phagocytosis as a pivotal therapeutic approach in cancer immunotherapy, especially addressing how intravital imaging sheds light on ADCP in a real-time manner. Moreover, various challenges for targeting phagocytosis, strategies to enhance ADCP, and factors limiting its efficiency have also been summarized. We also investigated the potential of chimeric antigen receptor (CAR) macrophages as a next-generation therapeutic modality that bridges the innate and adaptive immune systems to induce robust anti-tumor immune responses.

**Conclusions:**

The insights presented in this review will set the scene for future investigations of possible alternative approaches that consider the antibody-dependent cellular phagocytosis for improved cancer immunotherapy.

## Introduction

Macrophages are highly effective phagocytic cells that can ingest various substances, including pathogens, cellular debris, and dead cells [1]. Tumor-associated macrophages (TAMs) are found in large quantities in the tumor microenvironment (TME) [2, 3]. These cells play a crucial role in cancer progression, resistance to treatments, and metastasis [4–6]. TAMs can be categorized into two distinct subtypes, namely M1 and M2 macrophages, based on their polarization [7]. M1-polarized TAMs display a pro-inflammatory phenotype and form an immune environment that hinders tumor growth by producing pro-inflammatory cytokines, whereas M2-polarized TAMs typically exhibit an anti-inflammatory phenotype that supports an immunosuppressive environment, thereby promoting tumor growth by releasing anti-inflammatory cytokines and chemokines [8]. Both M1 and M2 TAMs can engulf cancer cells, though M1 macrophages are more effective [9, 10]. This phagocytic activity is primarily facilitated through the recognition of foreign particles, which is mediated by pattern recognition receptors (PRRs), scavenger receptors, Fc receptors, etc [11].

Cellular phagocytosis occurs via two primary mechanisms: efferocytosis, the engulfment of dead or dying cells, and ADCP. In ADCP, the binding of Fc gamma receptors (FcgRs) on macrophages to antibody fragment crystallizable (Fc) fragments triggers a crucial tumor-killing process that bridges innate and adaptive immune responses. Despite several studies on the mechanisms of phagocytosis, ADCP remains an underexplored strategy for the targeted elimination of malignant cells. This is partly due to a limited insight into how ADCP functions in vivo and the factors that affect the efficacy of mAbs in living organisms. Recently, intravital imaging has become a valuable real-time tool for providing critical insights into tumor biology. This technique enables the tracking of ADCP-inducing mAbs in the body and can help investigate how phagocytes clear mAb-opsonized target cells in the TME [12]. In this review, we will provide an overview of the current understanding of the cellular and molecular mechanisms underlying ADCP and explore how ADCP-inducing mAbs or ICIs treat cancer. Subsequently, we will explore the role of intravital imaging as a powerful tool for observing ADCP events in cancer immunotherapy. Finally, we will address some of the most pressing and unresolved questions in this field, offering insights into areas that necessitate further studies.

## ADCP via therapeutic antibodies in hematologic malignancies

**Fig. 1:**
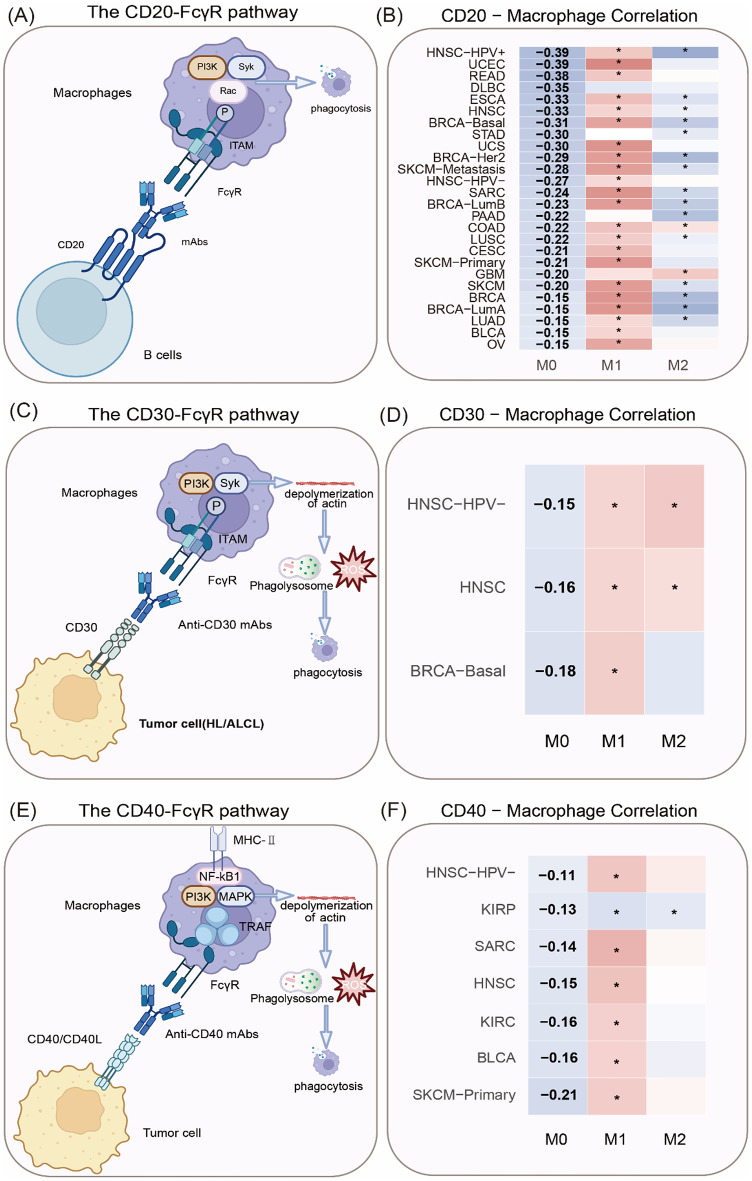
Signaling pathways involved in the function of various types of mAbs and ICIs (**A**) The CD20-FcγR pathway describes the interaction between the CD20 protein on B cells and FcγRs on immune effector cells. This interaction is essential for the therapeutic efficacy of anti-CD20 mAbs, which are used to treat B cell lymphomas and other diseases. The binding of anti-CD20 mAbs to CD20 on B cells activates this pathway, which subsequently recruits immune effector cells via their FcγRs, resulting in B cell depletion through various mechanisms. (**B**) TIMER immune infiltration analysis showed a negative correlation in the gene expression of total macrophages and CD20. (**C**,** E**) FcγR-dependent ADCP by macrophages plays a significant role in the efficacy of both anti-CD30 and anti-CD40 agents. (**D**,** F**) TIMER immune infiltration analysis deciphered a negative correlation in the gene expression of total macrophages with CD30 and CD40

Monoclonal antibodies have become a well-established approach and represent a substantial advancement in cancer treatment, mediating therapeutic efficacy not only through antigen-binding variable domains but also via the Fc domains [13]. The Fc domain interacts with its corresponding immunoglobulin FcR, a family of cell surface receptors expressed on various hematopoietic cells [13]. This family involves a series of receptors for IgG (FcgRI/CD64, FcgRII/CD32, and FcgRIII/CD16), IgE (FcϵRI), IgA (FcaRI/CD89), IgM (FcmR), and IgA/IgM (Fca/mR) [14, 15]. Within the human FcgR family, all Fc receptors, except for FcgRIIB, are activating receptors bearing immunoreceptor tyrosine-based activation motifs (ITAMs). They trigger activation after binding to IgG through intracellular ITAM multimerization (Fig. 1A) [16]. FcgRIIB is an inhibitory Fc receptor with an immunoreceptor tyrosine-based inhibition motif (ITIM) that inhibits the activation of ITAM-bearing immune receptors, thus exerting an immunosuppressive effect [16].

Studies have suggested that ADCP induced by resident macrophages in the liver or spleen is the primary mechanism responsible for mAb-mediated clearance of circulating B cells. Notably, many studies investigating the mechanisms and effector cells involved in ADCP cytotoxicity have focused on anti-CD20 mAb therapy, largely because therapeutic anti-CD20 mAbs have been clinically available for over 25 years, and their cytotoxic effects on circulating B cells can be easily evaluated [17]. ADCP in both normal and lymphoma-bearing mice necessitates the activation of FcγRs, particularly FcγRI, FcγRIII, and FcγRIV, on effector cells. Anti-CD20 treatment shows reduced efficacy in both non-tumor-bearing FcγR−/− mice and lymphoma-bearing FcγR−/− mice. In contrast, deficiency in the inhibitory receptor FcγRIIb prolongs the survival of lymphoma-bearing mice after treatment with anti-CD20 agents. Gong et al. investigated the role of Fc effectors by leveraging a mutant hCD20 mAb with two mutations in the Fc effector domain and found that the Fc mutation of hCD20 mAb impaired its binding to FcγRI, FcγRII, and FcγRIII and partly prevented complement activation. Treatment of hCD20-expressing mice with this mutant anti-hCD20-IgG2a mAb prevented mAb-mediated B-cell depletion in the circulation, lymph nodes, and splenic follicular zone [18]. Collectively, these findings underscore the critical role of FcγR activation and Fc effectors in regulating the efficacy of anti-CD20 therapies (Fig. 1A).

Previous studies have shown that macrophages are the primary effector cells involved in FcγR-dependent ADCP cytotoxicity during anti-CD20 immunotherapy (Fig. 1A). Mice with macrophage deficiency due to the Csf1^op/op^ mutation exhibit a reduced capacity to eliminate circulating and splenic B cells compared to control animals [19]. Likewise, macrophage depletion using clodronate liposomes was shown to compromise the efficacy of anti-CD20 treatment in both normal and lymphoma-bearing mice [20, 21]. Accumulating evidence highlighted the liver as a key site for the clearance of circulating B cells after treatment with anti-CD20 mAb [18, 22, 23]. For example, Gong et al. revealed that the ability of anti-CD20 mAbs to deplete circulating B cells was impaired in mice undergoing partial hepatectomy or in hCD20-expressing mice undergoing portal vein and celiac artery ligation [18]. In contrast, splenectomy did not affect the efficacy of anti-CD20 mAbs in depleting circulating B cells [18]. These findings suggest that macrophages, particularly those in the blood circulation, play a significant role as effector cells involved in anti-CD20 mAb therapy.

To better understand the correlation between gene expression of total macrophages and CD20 across various tumor types, our research group leveraged TIMER2.0 (http://timer.comp-genomics.org/), a computational tool commonly employed in cancer immunology, to investigate the relationship between gene expression profiles [24–26]. Notably, TIMER2.0 analysis does not involve ASV- or OTU-based microbial annotation. All immune-gene correlation analyses were conducted using the default Spearman rank correlation provided in the “Gene Module” of TIMER2.0. Also, TIMER2.0 employs preprocessed and normalized TCGA expression matrices and no additional manual filtering was applied. Finally, we selected the“purity-adjusted”option to control the tumor purity, which is a major confounder in immune deconvolution. The results indicated a negative correlation in the gene expression of total macrophages and CD20 across various tumors. Further investigation is required to elucidate whether CD20 directly modulates macrophage function or if this correlation reflects broader immunological shifts within the tumor microenvironment. Future studies will involve more in-depth functional assays, including flow cytometry and immunohistochemistry, to determine the localization and activation states of macrophages and B cells in tumor tissues. Additionally, exploring the specific cytokines and chemokines involved could provide more insight into how B cells, through CD20, might influence macrophage behavior. Understanding these interactions could have important implications for immunotherapy strategies targeting both B cells and macrophages in cancer.

## ADCP as a mechanism of action for other mAbs

**Fig. 2:**
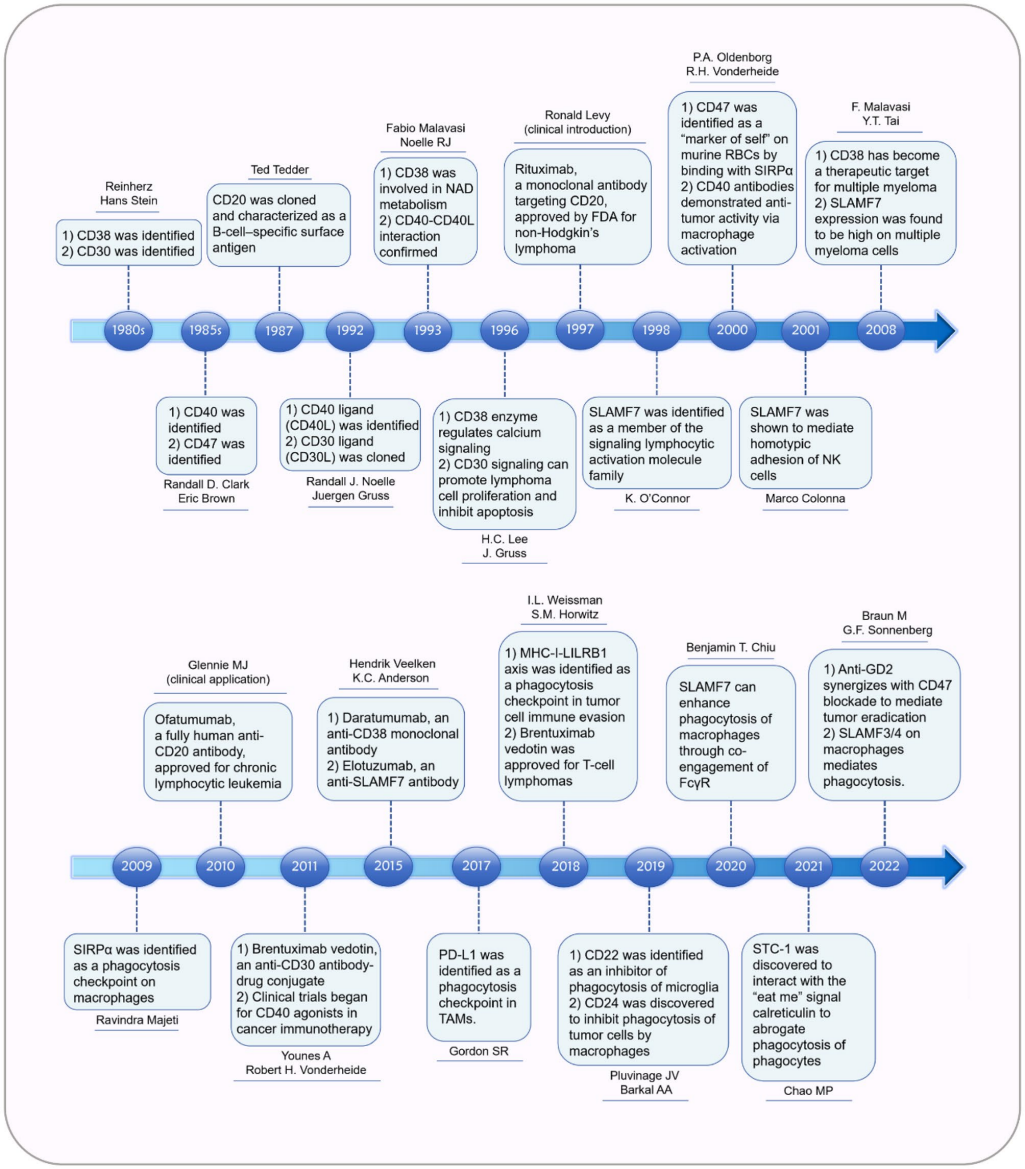
Timeline of mAb and ICI discovery. The discoverer and discovery time of various types of mAb and ICIs

Rituximab is the first genetically engineered chimeric murine-human anti-CD20 mAb approved for clinical use in 1997 (Fig. 2). It has become a cornerstone in the treatment of B-cell malignancies, significantly enhancing patients’ outcomes [13]. The success of rituximab has encouraged the development of a broad array of mAbs targeting diverse antigens, thereby advancing therapeutic strategies for both hematologic and solid tumors. For example, in the 2010s to 2015s, research into CD20 (Ofatumumab), CD38 (Daratumumab), CD30 (Brentuximab), CD40 mAb therapy for hematologic malignancies was evolving (Fig. 2) [27–30]. Daratumumab has been shown to enhance the phagocytosis of CD38 + malignant cells by TAMs. Anti-CD38 mAbs were shown to effectively treat Daudi-luciferase tumor and leukemic severe combined immunodeficiency (SCID)-beige xenograft models [30]. Moreover, FcγR-dependent ADCP by macrophages plays a significant role in the efficacy of both anti-CD30 and anti-CD40 agents (Fig. 1C-F) [28, 29]. In a disseminated Hodgkin disease model (L540cy), depletion of macrophages notably decreased the survival of anti-CD30-treated mice, whereas NK cell depletion did not affect the therapeutic outcome of anti-CD30 mAb [29]. Similarly, in a mouse model of non-Hodgkin lymphoma (NHL), macrophage depletion reduced the overall survival benefit of anti-CD40 agents, while depletion of neutrophils or NK cells did not affect the efficacy of anti-CD40 mAbs [28]. Collectively, as the number of clinically approved mAbs has grown exponentially since the introduction of rituximab, we are gaining substantial insights into how ADCP can be utilized as a cytotoxic strategy for immunotherapy.

## Phagocytosis checkpoints in cancer immunotherapy

**Fig. 3:**
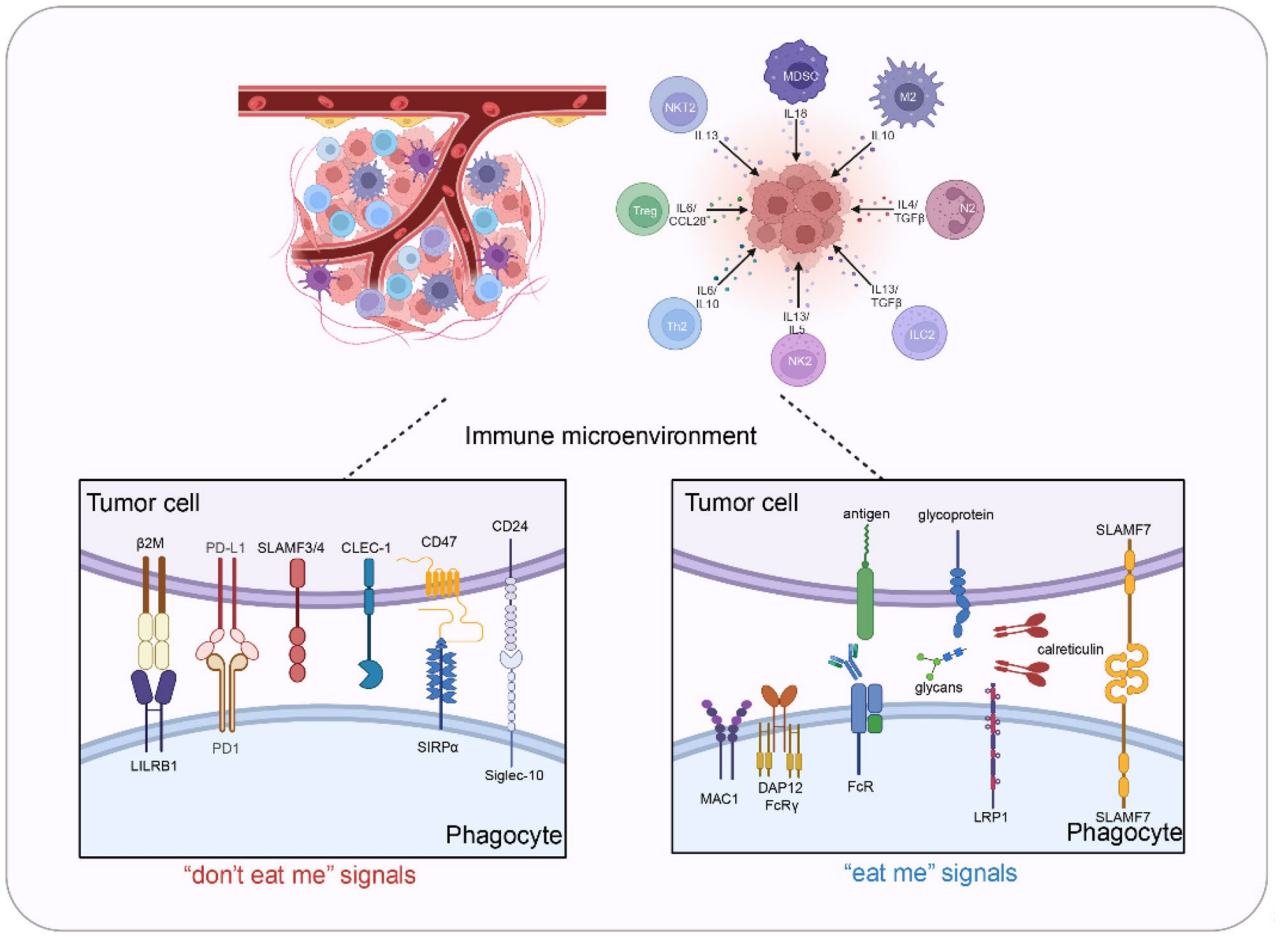
“don’t eat me” signals and “eat me” signals in the TME. Phagocytosis checkpoints, including CD47, CD24, β2M, GD2, STC-1, PD-L1, and SLAMF7, have become crucial targets in cancer immunotherapy as they function as “don’t eat me” signals or interact with “eat me” signals to inhibit immune responses

Innate immune checkpoints have been linked to decreased treatment efficacy in both hematologic malignancies and solid tumors, as they hinder the immune effector functions of macrophages, even though FcγR signaling pathways remain intact. Phagocytosis checkpoints, including CD47, CD24, β2M, GD2, STC-1, PD-L1, and SLAMF7, have become crucial targets in cancer immunotherapy as they function as “don’t eat me” signals or interact with “eat me” signals to inhibit immune responses (Fig. 3) [31]. These checkpoints play a key role in linking innate and adaptive immunity in cancer treatment. Genetic disruption of these checkpoints and inhibition of their signaling pathways significantly enhances phagocytosis and reduces tumor growth. Among these checkpoints, CD47 is the most extensively studied and is considered a promising target for cancer treatment. CD47 antibodies and inhibitors have been explored in a subset of preclinical studies and clinical trials, as depicted in Tables 1, 2 and 3 [31].

### The CD47-SIRPα signaling pathway

**Fig. 4:**
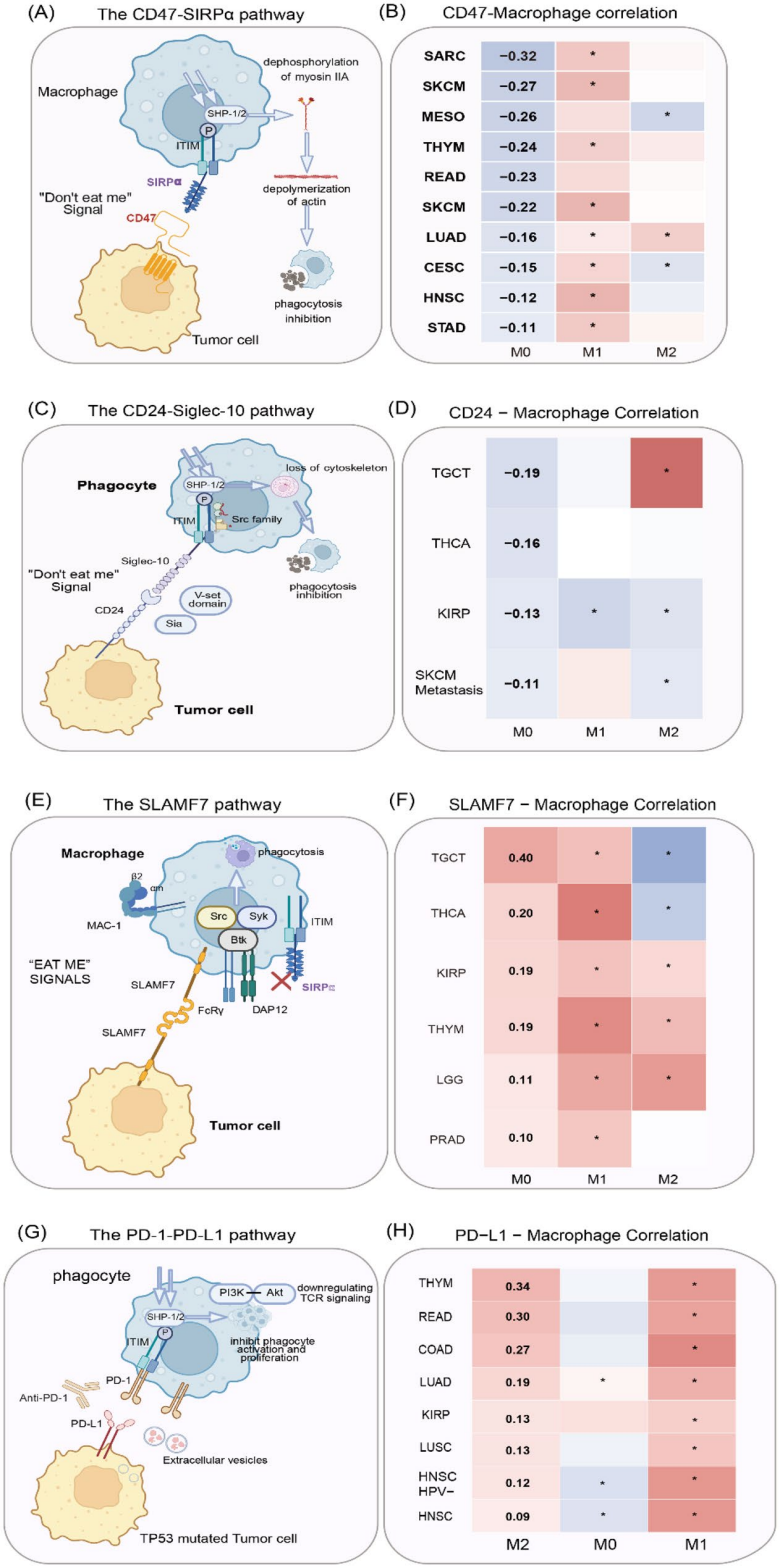
ICIs-mediated ADCP in cancer immunotherapy. (**A**) CD47 on tumor cell surfaces binds to SIRPα on the macrophage membrane, triggering the phosphorylation of the ITIM motif in SIRPα by Src family kinases, SHP-1 and SHP-2. This recruitment leads to the phosphorylation of myosin IIA, which subsequently inhibits the function of non-muscle myosin IIA. Dephosphorylation of myosin IIA in macrophages leads to actin depolymerization, thereby inhibiting phagocytosis. (**B**) TIMER immune infiltration analysis exhibited a negative correlation in the gene expression of total macrophages and CD47. (**C**) The inhibitory receptor Siglec-10 on macrophage surfaces binds to its ligand CD24 on cancer cells, triggering an ITIM or ITIM-like motif within the cytoplasmic domain of Siglec-10 to interact with Src family kinases. These kinases then phosphorylate the ITIM tyrosine, which subsequently recruits SHP-1/SHP-2. SHP-1 specifically binds to the phosphorylated ITIM domain and dephosphorylates it, which results in cytoskeletal remodeling and inhibition of phagocytosis. (**D**) TIMER immune infiltration analysis indicated a negative correlation in the gene expression of total macrophages and CD24. (**E**) SLAMF7 on macrophages binds to MAC-1, which interacts with FCRγ and DAP12 for the recruitment of Src family kinases, including Syk and Btk, thereby enhancing phagocytosis. The binding of SLAMF7 on macrophages to SLAMF7 on phagocytes is essential for the phagocytosis of hematological cancers. (**F**) TIMER immune infiltration analysis showed a positive correlation in the gene expression of total macrophages, M1 macrophages with SLAMF7. (**G**) Inhibition of the PD-1-PD-L1 signaling pathway with either a PD-1 or a PD-L1 inhibitor enhanced macrophage-mediated phagocytosis. (**H**) TIMER immune infiltration analysis revealed a positive correlation in gene expression between M2 macrophages and PD-L1

CD47 is widely expressed on the surface of various cancer cells and functions as a critical inhibitor of phagocytosis, enabling tumor cells to evade immune surveillance by the host. CD47 has been identified as a promising therapeutic target due to its role in preventing the phagocytosis of tumor cells (Fig. 4A). The interaction between CD47 on tumor cells and SIRPα on phagocytes triggers ITIM phosphorylation in SIRPα by Src family kinases, including SHP-1 and SHP-2, thereby inhibiting phagocytosis (Fig. 4A). Targeting the interaction of CD47 and SIRPα disrupts their binding and enhances the ability of macrophages to remove cancer cells by promoting cytokine secretion and enhancing the immune response. In addition, targeting this interaction facilitates tumor cell elimination via NK-cell-mediated antibody-dependent cellular cytotoxicity (ADCC) and prompts direct tumor cell removal. Moreover, targeting CD47 enhances the capacity of dendritic cells (DCs) to phagocytize tumor cells and present tumor antigens to T cells, further promoting the anti-tumor immune response.

Hu5F9-G4 was the first humanized antibody developed to target CD47, initially used in pediatric patients with primary brain malignancies [32]. This antibody has been administered for the treatment of five distinct types of childhood brain tumors and holds potential for the treatment of various malignant tumors of the central nervous system (CNS). Another antibody, SIRPαD1-Fc, a novel fusion protein targeting CD47, promotes autophagy in non-small cell lung cancer (NSCLC) cells by inhibiting the Akt/mTOR pathway and enhancing the production of reactive oxygen species (ROS). Combining CD47-SIRPα targeting with other therapeutic approaches may improve treatment outcomes. Common combination strategies involve the use of additional therapeutic antibodies, macrophage recruitment, and a combination of chemotherapy and radiotherapy to augment the efficacy of ADCP-inducing antibodies and inhibit tumor metastasis. For instance, inhibition of the CD47-SIRPα pathway alongside treatment with sodium stibogluconate (SSG) ameliorated the resistance of anti-CD20-opsonized B-cell lymphoma to neutrophil-mediated killing [32]. Hu5F9-G4 is currently undergoing phase II/III clinical trials for the treatment of acute myeloid leukemia (AML) and has shown a favorable safety profile when combined with azathioprine (AZA). Preliminary results have indicated that the combination of magrolimab and AZA exhibits promising efficacy in patients with AML and mutated TP53 [33, 34]. 

Finally, we employed TIMER2.0 to assess the correlation between gene expression in total macrophages and CD47. The analysis revealed a significant negative correlation in the expression of CD47 and total macrophages across various tumor types. This finding suggests a potential modulatory relationship between macrophage polarization and CD47 expression, warranting further exploration to uncover the underlying molecular mechanisms driving this association.

### The CD24-Siglec-G/10 signaling pathway

CD24 is commonly overexpressed in B-cell lymphomas, hepatocellular carcinoma (HCC), gliomas, small cell lung cancer (SCLC), and breast cancer [35]. It has been identified as a marker of both cancer diagnosis and prognosis. CD24 plays a key role in mediating cell adhesion, interactions with substrates, and other processes, such as cell recognition, activation, signal transduction, proliferation, differentiation, extension, and movement [36]. Recent studies have increasingly shown that CD24 has a significantly higher expression level on the tumor cell membrane than in adjacent tissues. CD24 overexpression was also shown to be positively correlated with tumor development and progression. High levels of CD24 on tumor cells not only promote tumor progression by affecting cell proliferation and migration but also enable tumor cells to evade immune cell-mediated destruction through interactions with immune cells in the TME. The binding of CD24 on tumor cells to Siglec-10 on immune cells triggers inhibitory signaling pathways mediated by SHP-1/SHP-2, which facilitates immune evasion by suppressing T and NK cell-mediated killing and macrophage-mediated phagocytosis (Fig. 4C). Recent studies have shown that inhibiting the interaction between CD24 and Siglec-10 using a CD24 inhibitor or via gene editing can significantly promote the recognition of CD24-expressing tumor cells by macrophages. For example, Wang et al. found that CD24 expression was significantly elevated in TNBC cells with high potential of lung and lymph node metastasis [37]. Depletion of CD24 inhibited the growth of primary tumors, prevented metastasis to the lymph nodes and lungs, and decreased the number of blood and lymphatic vessels in the TME. CD24 knockdown impaired EGFR/Met-mediated signaling and lowered the levels of molecules associated with lymphangiogenesis and angiogenesis by inducing EGFR and Met protein instability through the lysosomal degradation [37]. Treatment with a CD24 mAb reduced lung metastasis and extended the survival of mice with lung metastasis. Finally, we explored the gene expression correlation between total macrophages and CD24 using TIMER2.0. As expected, the analysis revealed a negative correlation between the gene expression of total macrophages and CD24 across several tumors (Fig. 4D). These longitudinal data suggest that CD24 may be a major target in exploring novel checkpoints for the phagocytosis of cancer cells.

### The ‘eat me’ signal SLAMF7

‘Eat me’ signals are molecules released from or expressed on cells and promote phagocytosis. Although most ‘eat me’ signals are located on the cell surface, some may be released extracellularly and subsequently bind to the target cell. Notable examples of ‘eat me’ signals include the lipid phosphatidylserine, the intracellular adhesion molecule ICAM-3, calreticulin, cell surface-bound thrombospondin, annexin I, oxidized low-density lipoprotein, complement factors, and various glycosylation alterations on apoptotic cells. These signals have been extensively reviewed in previous studies.

SLAMF7, also referred to as CRACC, CD319, or CS1, is a member of the SLAM receptor family that is found on both tumor cells [38–40] and immune cells, such as activated CD4 and CD8 T cells, dendritic cells (DCs), NK cells, and B cells. SLAMF7 on macrophages binds to the corresponding SLAMF7 on hematopoietic cells to facilitate phagocytosis (Fig. 4E). Macrophages lacking SLAMF7, but not those deficient in other signaling lymphocytic activation molecule family receptors (SFRs, e.g., SLAMF1-6), show impaired phagocytic activity. SLAMF7 on macrophages also interacts with integrin macrophage-1 antigen (MAC-1). MAC-1 is expressed on macrophages and promotes the phagocytosis of cancer cells (Fig. 4E). MAC-1 interacts with ITAM via a complement receptor (CR3) consisting of the α-subunit CD11b (αm) and β-subunit of CD18 (β2) [40], FcRγ, and DAP12 to activate immune cells through signaling pathways involving Src, Syk, and Bruton’s tyrosine kinase (Btk), thereby enhancing phagocytosis via IgG-mediated FcR pathway (Fig. 4E) [41]. Hence, the expression of MAC-1 on macrophages is essential for SLAMF7-mediated phagocytosis of tumor cells (Fig. 4E). Chen et al. demonstrated that the phagocytosis of hematopoietic tumor cells is heavily dependent on SLAMF7 following the inhibition of the CD47-SIRPα axis [40]. However, their study presented only two B-cell lines as examples of susceptible SLAMF7-positive cells, with diffuse large B-cell lymphoma (DLBCL) proposed as a potential target for CD47 blocking therapy solely based on high SLAMF7 mRNA expression in a patient cohort. In contrast, He et al. employed the same antibody clone as Chen et al. and found that only one out of seven DLBCL cell lines expressed detectable levels of cell surface SLAMF7. Notably, no SLAMF7 surface expression was observed on primary patient-derived leukemic DLBCL or mantle cell lymphoma (MCL) cells [42]. Furthermore, both the F(ab′)2 fragment of the CD47 antibody inhibrix and the full antibody inhibrix induced significant phagocytosis in all tested DLBCL lines. In line with these findings, there was no correlation between SLAMF7 expression and phagocytosis induced by CD47 inhibitor treatment in a cohort of DLBCL and non-Hodgkin lymphoma (NHL) cell lines [42].These results thus clearly contrast with the conclusions drawn by Chen et al., suggesting that SLAMF7 is not essential for CD47-mediated phagocytosis. Given these discrepancies, further investigations are warranted to clarify the role of SLAMF7 in macrophage-mediated phagocytosis.

Likewise, Egmond et al. explored macrophage-mediated ADCP as the mechanism by which elotuzumab kills tumor cells. Elotuzumab is a monoclonal antibody targeting the SLAMF7 receptor in multiple myeloma. M1 macrophages cocultured with myeloma cells exhibited enhanced ADCP following treatment with elotuzumab. The administration of an Fc-inert variant of elotuzumab significantly diminished its survival benefits in the xenograft mouse models of multiple myeloma [22]. Additionally, depletion of macrophages or NK cells in EG7-hSLAMF7 tumor-bearing immunocompetent mice treated with Elotuzumab-IgG2a increased tumor burden compared to mice treated with Elotuzumab-IgG2a alone. However, a similar tumor burden was observed for NK cell-depleted mice and macrophage-depleted mice, indicating that both NK cells and macrophages are essential for the therapeutic efficacy of elotuzumab. Finally, we investigated the correlation of gene expression between total macrophages and SLAMF7 using TIMER2.0. As expected, the analysis revealed a positive correlation in gene expression between total macrophages and SLAMF7 across several tumors (Fig. 4F**)**.

### The PD-1-PD-L1 signaling pathway

In recent years, PD-1/PD-L1 immune checkpoint blockade has become a robust clinical strategy for cancer treatment. PD-1 is an inhibitory immune modulatory receptor expressed not only in T cells in peripheral tissues but also in NK cells, DCs, and B cells [41, 42]. PD-1 is notably upregulated on tumor-specific T cells. When PD-1 interacts with its ligand PD-L1, it induces immune tolerance and T cell exhaustion within the TME. Blockade of the PD-1 pathway has been verified to restore the function of exhausted T cells, leading to dramatically anti-tumor activity. In a MC38 mouse model, Dahan et al. demonstrated that FcγRs diminished the anti-tumor efficacy of a PD-1 inhibitor by the elimination of CD8 + tumor-infiltrating lymphocytes via antibody-dependent cellular cytotoxicity [43]. Furthermore, the engagement of FcγRIIb by a PD-1 inhibitor can also reduce its anti-tumor activity. Arlauckas et al. showed that anti-PD-1 antibodies are seized from the T-cell surface by FcγR-bearing macrophages. Blocking FcγRs, therefore, extends the duration of anti-PD-1 antibody binding to CD8 + TILs, thus enhancing anti-tumor activity [44]. A preclinical study conducted by Chen.et.al also indicated that binding to FcγRI negatively impacts the anti-tumor efficiency of anti-PD-1 antibodies in a humanized xenograft mouse model [45]. This binding facilitates the phagocytosis of PD-1 + T cells by FcγRI+ macrophages via ADCP, effectively reversing the antibody’s function from blocking to activation [46].

Recently, several studies have documented that the frequency of hyperprogression in a subset of cancer types treated with FDA-approved PD-1 inhibitor is significantly higher than in chemotherapy controls [47, 48]. The interaction between FcγR+ macrophages and PD-1 inhibitors in NSCLC during PD-1 blockade therapy was also noted by Lo Russo et al. [49]. These findings underscore the need for an anti-PD-1 antibody with pure blocking activity, as antibodies that bind FcγRs can mediate cross-linking between FcγR+ macrophages and PD-1 + T-cells, leading to the depletion of PD-1 + T effector cells and compromising T-cell-mediated tumor inhibition [45].

At present, some mAb isotypes are waiting for FDA approval or being investigated in clinical trials. For example, penpulimab, a mutated IgG1 anti-PD1, is approved by the Chinese National Medical Products Administration (NMPA) for the targeting of Hodgkin’s lymphoma and NSCLC. Penpulimab was developed with a modified Fc region to completely block binding to FcγR, thereby preventing ADCP [50]. Another example is balsitilimab, an anti-PD1 mAb currently being tested in combination with new anti-CTLA4 mAbs for various cancers, such as orzalifrelimab (for cervical cancer) [51] and botensilimab (for metastatic colorectal cancer) [52]. The Fc region of these antibodies has been engineered to enhance ADCC for anti-CTLA4 while preventing it for anti-PD1.

Although the role of the PD-1-PD-L1 axis in regulating T cell immune responses is well-established, recent studies have highlighted its involvement in the phagocytic activity of TAMs [53]. PD-1 is expressed not only in T cells in peripheral tissues but also in NK cells, DCs, and B cells [54, 55]. TAMs exhibit higher levels of PD-1 compared to circulating monocytes or splenic macrophages, with PD-1 expression increasing with tumor volume after engraftment. Moreover, PD-1 can facilitate the polarization of macrophages toward the M2 phenotype. The majority of PD-1 + TAMs exhibit M2-like characteristics, which are considered to be pro-tumorigenic in the TME [56, 57].

Studies on bone marrow transplantation have shown that the majority of PD-1 + TAMs originate from circulating leukocytes, rather than resident immune cells [53]. PD-1 + TAMs possess decreased phagocytic capacity compared to PD-1 − TAMs, suggesting that PD-1 expression on TAMs undermines their phagocytic activity. Inhibition of the PD-1-PD-L1 signaling pathway with either a PD-1 or a PD-L1 inhibitor enhanced macrophage-mediated phagocytosis and improved the survival of NOD SCID gamma (NSG) mice without T cells (Fig. 4G) [53]. Additionally, tumor cells with TP53 mutations release high levels of extracellular vesicles and exhibit reduced macrophage-mediated phagocytosis. However, inhibiting PD-L1 on the extracellular surface of TP53-mutant cells restores the phagocytic function of macrophages, suggesting the critical role of the PD-1-PD-L1 axis in regulating macrophage-mediated phagocytosis in TP53-mutated cancers [58]. The PD-1/PD-L1 axis directly affects macrophages in the tumor tissue, suggesting that PD-1 not only suppresses cytotoxic T-cell activity but also inhibits macrophage-mediated phagocytosis, uncovering a novel mechanism by which the PD-1/PD-L1 axis regulates macrophage-mediated phagocytosis. Moreover, Lipopolysaccharide (LPS)-induced Toll-Like Receptor 4 (TLR4) signaling upregulates PD-1 expression in macrophages, and PD-L1 binding to PD-1 on these macrophages promotes the polarization of tolerogenic Signal Transducer and Activator of Transcription 6 (STAT6)-dependent macrophages, finally facilitating tumor growth [59].

The expression of PD-L1 on macrophages activates constitutive signaling, which inhibits macrophage activation and proliferation by suppressing the mechanistic target of rapamycin (mTOR) signaling pathway [31]. Macrophages deficient in PD-L1 (PD-L1−/−) exhibit enhanced activation and proliferation. Treatment with PD-L1 antibodies can increase the production of spontaneous proinflammatory cytokines and costimulatory molecules. Mechanistically, PD-L1 blockade enhances the expression of costimulatory molecules (CD86 and MHC-II) and promotes the secretion of proinflammatory cytokines (TNFα and IL-12), consistent with the characteristics of M1 macrophages [31]. However, during metabolic reprogramming, PD-L1 promotes M2 polarization through the Erk/Akt/mTOR signaling pathway [31].

Finally, we also investigated the gene expression correlation between total macrophages and PD-L1 using TIMER2.0. The analysis revealed a positive correlation in gene expression between M2 macrophages and PD-L1 across several tumors (Fig. 4H). These comprehensive and longitudinal data indicate that PD-L1 is a prominent target in investigating novel checkpoints controlling the phagocytosis of tumor cells.

**Fig. 5:**
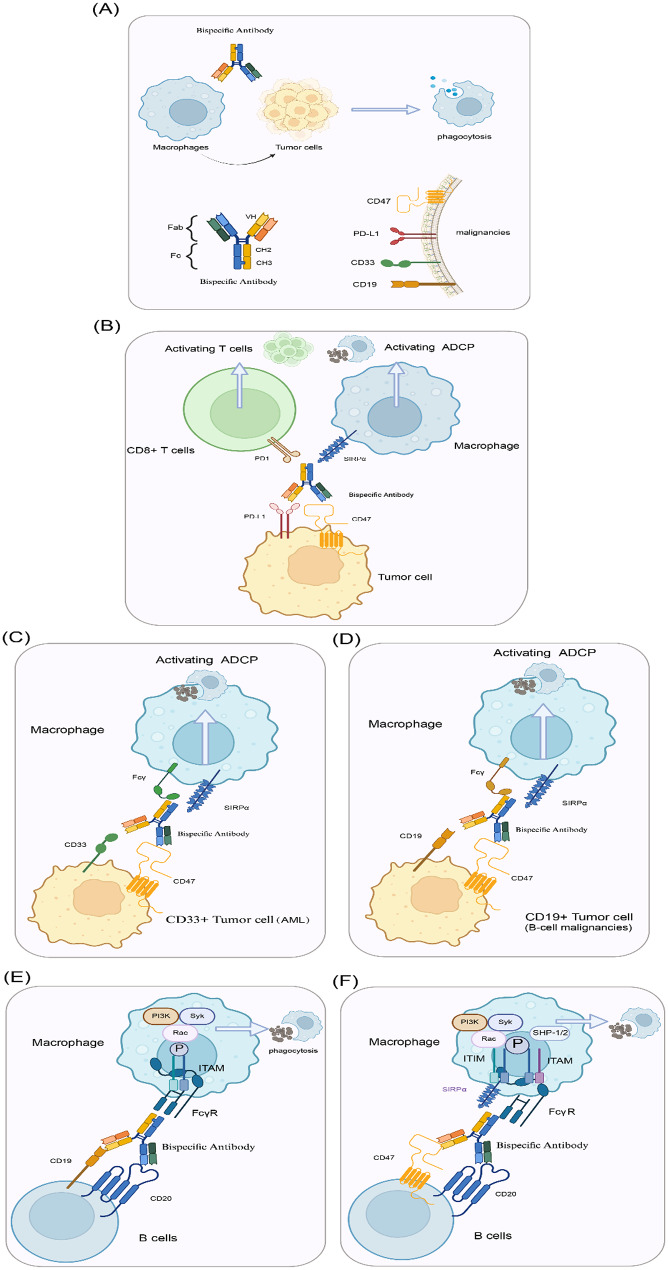
Current and potential bispecific antibodies in hematological malignancies. (**A**) hematological malignancies can be treated with bispecific antibodies that bind “don’t eat me” signal molecules expressed by macrophages and markers specific to tumor cell. (**B-F**) CD47/PD-L1, CD33/CD47, CD47/CD19, CD19/CD20, and CD47/CD20 bispecific antibodies were designed with the IgG1 isotype to trigger the macrophage-mediated ADCP response

## Systematic activations of macrophages through bispecific antibodies

With the growing recognition of tumor heterogeneity, bispecific antibodies have emerged as a promising therapeutic approach for cancer. Although much attention has been paid to targeting T cells or NK cells with bispecific antibodies [60, 61], the application of this strategy to macrophages has received less attention. To our knowledge, the idea of using macrophages as tumoricidal effectors through bispecific antibodies was first introduced in 2015 [1]. So far, only a few bispecific antibodies targeting macrophages have been developed, a small proportion of which have been validated in clinical trials.

The involvement of CD47 in immune evasion and the therapeutic potential of CD47 inhibitors were initially demonstrated in an AML model [62, 63]. Building on this, HMBD004, a bispecific antibody targeting CD47 and CD33, was developed by combining an anti-CD33 gemtuzumab with a humanized anti-CD47 antibody, utilizing a 1 + 1 IgG format (Fig. 5C). This bispecific antibody effectively blocked the CD47-SIRPa axis and induced phagocytosis without causing significant hemagglutination of erythrocytes in vitro. Moreover, in xenograft mouse models of AML, treatment with HMBD004 markedly reduced tumor burden and prolonged progression-free survival (PFS). NI-1701 is a CD47/CD19 bispecific antibody designed with the IgG1 isotype to trigger an ADCP response (Fig. 5D) [64]. This antibody exhibited potent in vitro and in vivo activity against various types of B cell malignancies in experimental models. Simultaneous engagement of CD47 and CD19 on B cells is necessary to induce robust ADCP of target cells [64, 65].

Considering the efficacy of the anti-CD47 antibody magrolimab (hu5F9-G4) in combination with rituximab for treating B-cell non-Hodgkin’s lymphoma, a rational bispecific antibody design approach is pairing the anti-CD47 component with an additional arm that targets a B-cell-specific antigen, such as CD19 [64–66] or CD20 (Fig. 5E-F) [67, 68]. Two bispecific antibodies have recently been described utilizing this approach: (1) incorporating anti-CD20 variable domains at the N-terminal of the anti-CD47 antibody’s variable domain; and (2) fusing the N-terminal V-set Ig domain of SIRPa with the N-terminal of the heavy-chain variable domain of a CD20 mAb. Both bispecific antibodies exhibited enhanced tumor cell killing without causing on-target off-tumor effects. Moreover, they showed superior in vivo efficacy in Raji xenograft models, prolonging survival compared to monotherapy or combination therapy. In 2017, RTX-CD47, a novel bispecific antibody, was designed to target both CD47 signaling and CD20-positive cells. This was achieved by fusing the CD20-targeting scFv fragment from rituximab with a CD47-blocking scFv [69].

In addition to targeting tumor antigens, there is a growing interest in simultaneously targeting multiple immune checkpoints, such as PD-L1 and CD47 (Fig. 5B). PD-L1 is often overexpressed in tumor tissues, and its blockade may enhance the retention of a CD47/PD-L1 bispecific antibody in tumor tissues. Beyond its role as a well-known T cell checkpoint [54], PD-L1 serves as a “don’t eat me” signal for macrophages [53]. Therefore, PD-L1 blockade can restore the anti-tumor functions of TAMs, and CD47/PD-L1 bispecific antibodies enhance ADCP. A recent proof-of-principle study evaluated the anti-tumor efficacy of this bispecific antibody in several mouse models [70, 71]. Compared to monotherapy with anti-CD47 or anti-PD-L1 or their combination, the CD47/PD-L1 bispecific antibody, which simultaneously targeted both antigens on tumor cells, more effectively inhibited tumor growth and prolonged the survival of recipients [70, 71]. Mechanistically, the systemic administration of the dual-targeting agent substantially enhanced the anti-tumor T cell response, DNA sensing, and dendritic cell cross-presentation [70, 71].

**Table 1:** Phagocytosis checkpoints and working mechanisms

mAb and ICIs	Expression and mechanisms	Mouse models	Reference
Chimeric anti-CD20 MoAb IDEC-C2B8	Overexpressed on B cells and promote phagocytosis	Relapsed low-grade B-cell lymphoma	[17]
Anti-hCD20-IgG2a mAb	Overexpressed on B cells and mAb-mediated B-cell depletion	Human CD20+ models	[18]
Anti-CD20 mAbs	Overexpressed on B cells and Fc Receptor–dependent ADCP cytotoxicity	Lymphoma-bearing mice	[19]
Anti-CD20 mAbs	Clodronate-mediated depletion of macrophages eliminated the therapeutic benefit of CD20 mAb	A mouse lymphoma model	[20]
SGN-40 (CD40)	Highly expressed on B-cells, DCs, monocytes, TAMs and activated B-cells; Fc–Fc*γ*R interaction mediated ADCP	Non-Hodgkin’s lymphoma	[28]
SGN-30 (CD30)	Anti-tumor activity of SGN-30 by inducing cellular cytotoxicity via ADCP	A disseminated model of Hodgkin disease	[29]
Daratumumab (CD38)	Anti-tumor activity of Daratumumab by inducing cellular cytotoxicity via ADCP	Leukemic xenograft mouse models	[30]
Hu5F9-G4 (Magrolimab)	Overexpressed on cancer cells; Fc Receptor–dependent ADCP cytotoxicity	Medulloblastoma leptomeningeal models	[32]
Hu5F9-G4	Overexpressed on cancer cells; Fc Receptor–dependent ADCP cytotoxicity	Acute myeloid leukemia	[33]
CD47	Overexpressed on glioblastoma cells; Fc Receptor–dependent ADCP cytotoxicity	Glioblastoma	[72]
CD47	Highly expressed in ovarian cancer; Fc Receptor–dependent ADCP cytotoxicity	Ovarian cancer	[73]
CD24	Highly expressed in lung and lymph node metastatic triple negative breast cancer (TNBC) cells; immune evasion via CD24-Siglec-G/10 signaling pathway	TNBC	[37]
CD24	CD24 positive cells contribute to the breast cancer heterogeneity	Breast cancer	
Elotuzumab (SLAMF7)	Highly expressed on myeloma and other cells of hematopoietic lineage including NK cells; mediated by Fc-FcγR interaction of elotuzumab with macrophages	Immunocompromised xenograft models of multiple myeloma	[27]
PD-L1	PD-1-PDL-1 axis in regulating macrophage phagocytosis in TP53-mutated cancers	TP53-deficient B-cell lymphomas	[58]

Abbreviations: TNBC, triple -negative breast cancer; DCs, dendritic cells

**Table 2:** Bispecific checkpoints and working mechanisms

Bispecific Antibody	Expression and mechanisms	Tumor model	Reference
Anti-CD47×CD19 (NI-1701)	Overexpressed on B cells and promote phagocytosis by ADCP	B cell malignancy models	[64]
Anti-CD47×CD19	Overexpressed on B cells and promote phagocytosis by ADCP	B cell malignancy models	[65]
Anti-CD47×CD19	Overexpressed on B cells and promote phagocytosis by ADCP	Burkitt’s lymphoma	[66]
Anti-CD47×CD20	Overexpressed on B cells and promote phagocytosis by ADCP	Non-Hodgkin lymphoma (NHL)	[74]
Anti-CD47×CD20	Promote phagocytosis by ADCP	Non-Hodgkin lymphoma (NHL)	[68]
Anti-CD47×PDL1	Induce phagocytosis of tumor cells and medicate ADCC	Immune-competent mouse model of MC38	[70]
Anti-CD47×PDL1	Highly expressed on tumor cells and promote phagocytosis	MC38 mouse model	[71]
Anti-CD20/MerTK (CD20/18G7-LALAPG)	Induce antigen-specific target cell via activation of MerTK in TAMs	N/A	[75]
Anti-Aβ/MerTK (3D6/20F5-LALAPG)	Enhance Aβ aggregate clearance by microglial cells	N/A	[75]
Anti-CD70/SIRPα	High expression in B cell malignancies	Burkitt’s lymphoma xenograft	[76]

Abbreviations: NHL, Non-Hodgkin lymphoma; TAMs, Tumor associated macrophages; N/A, Not applicable

**Table 3:** Clinical tries of immune checkpoint inhibitors

Drug	Main component	Target	Disease	Clinical phase	National Clinical Trial
AK117	Monoclonal Antibody	CD47	MDS	Phase1/2	NCT04900350
			AML	Phase1/2	NCT04980885
ALX	Fusion protein	CD47	Gastric Cancer	Phase2/3	NCT05002127
			NHL	Phase1/2	NCT05025800
SGN-CD47M	Antibody–Drug Conjugates	CD47	Solid Tumor	Phase 1	NCT03957096
IC9-GD2 T cells	CAR-T	GD2	Neuroblastoma	Phase1	NCT01822652
Ch14-18/CHO	Monoclonal Antibody	GD2	Neuroblastoma	Phase1	NCT02914405
Hu14.18-IL2	IL-2 linked to hu14.18 mAb	GD2	Melanorma	Phase2	NCT03958383
OSE-172	Monoclonal antibody	SIRPα	Solid Tumor	Phase1	NCT03990233
Anti-SIRPα	CC-95,251	SIRPα	Advanced Solid and Hematologic Cancers	Phase1	NCT03783403
BND-22	Monoclonal Antibody	LILRB1	Advanced Solid Tumors	Phase 1/2	NCT04717375
TTX-080	Monoclonal Antibody	HLA-G	advanced refractory / resistant solid malignancies	Phase 1	NCT04485013
CD24Fc	CD24 Extracellular Domain-IgG1 Fc Domain Recombinant Fusion Protein CD24Fc	CD24	solid tumors	Phase 1/2	NCT04552704
			Metastatic Melanoma	Phase Ib/II	NCT04060407
BAT7104	bispecific antibody	CD47/ PD- L1	Solid Tumor	Phase 1	NCT05200013
RRx-001	Small molecular	CD47/ SIRPα axis	Small Cell Lung Cancer	Phase 3	NCT03699956
			Colorectal Neoplasms	Phase 2	NCT02096354
			Oral Mucositis	Phase 2	NCT03515538
IMM2902	bispecific antibody	CD47/ SIRPα	HER2-expressing Advanced Solid Tumor	Phase 1	NCT05076591
IMM0306	bispecific antibody	CD47/ CD20	B-NHL	Phase 1	NCT04746131
SG2501	bispecific antibody	CD38/ CD47	Hematological Malignancy	Phase 1	NCT05293912
IBI322	bispecific antibody	CD47/ PDL1	Advanced Malignancies	Phase 1	NCT04338659/ NCT04328831
			Hematological Malignancy	Phase 1	NCT04795128
Pembrolizumab + ALX148	blocking PD-1 and CD47	CD47/ PDL1	Ovarian Cancer	Phase 2	NCT05467670
JMT601	Fusion protein	CD20/ CD47	NHL	Phase 1/2	NCT04853329
DSP107	bispecific antibody	CD47/ 41BB	NSCLC	Phase1/2	NCT04440735
SL-172,154	bispecific antibody	SIRPα/ CD40L	SCC	Phase 1	NCT04502888
TG-1801	bispecific antibody	CD47/ CD19	Hematological Malignancy	Phase 1	NCT04806035
HX009	bispecific antibody	CD47/ PD1	Lymphoma	Phase 2	NCT05189093
NGM707	Dual antibody	LILRB1/ LILRB2	Advanced or Metastatic Solid Tumor Malignancies	Phase 1/2 Phase 1/3	NCT04913337/ NCT04913337

## Applications of intravital imaging in studies on ADCP

The integration of intravital microscopy in monitoring ADCP events holds promise for predicting the therapeutic efficiency of anti-CD20 agents in B malignancies and various autoimmune disorders. However, much less is known about the precise mechanisms through which anti-CD20 antibodies facilitate B cell depletion in vivo. Specifically, the anatomical sites involved, the effector cell types, and the underlying mechanisms of action remain largely unknown. In this context, Bousso. et al. identified the liver as a major site for B cell depletion and revealed that the marked decrease in B cells in secondary lymphoid organs is predominantly due to recirculation [21]. Using intravital imaging, their data showed that KCs play a crucial role in the rapid arrest and phagocytosis of B cells in the liver sinusoids after treatment with anti-CD20 agents [21]. The aforementioned findings highlight ADCP mediated by KCs as a key mechanism of anti-CD20 therapy and provide a basis for further optimization of the therapeutic efficacy of mAb [21]. Likewise, Yang et al. visualized the phagocytosis of NAD(P)H^hi^ CD47-AF488 + cancer cells by F4/80-PE macrophages in a skinfold model of triple-negative breast cancer during immunotherapy with anti-CD47 mAb. Such findings offer robust in vivo evidence supporting the role of CD47 blockade in ADCP [12]. In a recent study, three distinct anti-EGFR mAbs, namely panitumumab, zalutumumab, and cetuximab, were equally effective in opsonizing tumor cell lines in vitro. Furthermore, intravital imaging indicated that all three mAbs facilitated ADCP in cancer cells via KCs [77].

Despite the fact that intravital imaging offers numerous benefits for studies of biological samples at high spatial and temporal resolutions with limited photo-damage and superior tissue penetration, it has several inherent limitations. First, although fluorescence labeling and second-harmonic generation (SHG) enable the visualization of target cells and tissues, not all regions of interest containing cellular information can be visualized with multiphoton microscopy [12]. Consequently, observations should be interpreted cautiously to prevent misinterpretation. Second, while multiphoton microscopy allows for deeper tissue penetration compared to conventional confocal microscopy, it is limited by a maximum depth of 200 μm in hard tissues (e.g., bone) and approximately 800-1,000 μm in soft tissues (e.g., cerebral cortex) [78].As a result, it is mainly applicable to small animal such as mice and rats. Furthermore, due to substantial light scattering by the skin, internal organs must be exteriorized. This requires surgical intervention and alteration of oxygen and humidity levels, which may potentially impact cellular activity [79]. Innovations in optical systems and fluorochrome, such as enhanced light emission and resolution, are essential to solve these problems.

## Potential challenges for targeting phagocytosis

To date, phagocytosis checkpoints have garnered increasing attention, with substantial progress made in studying “don’t eat me” signals. Over time, more and more phagocytosis checkpoints have been identified, and clinical evaluations of these targets are underway. CD47-SIRPα, the first identified phagocytosis checkpoint, has entered clinical trials (See Table 3). Targeting CD47 leads to fewer toxic adverse events than other therapeutic approaches. It facilitates the complete engulfment of cancer cells by macrophages while minimizing the release of cellular contents upon cell death. Disrupting the interaction between CD47 and SIRPα has emerged as a promising approach for immunotherapy and treatment of advanced cancers, which can enhance the ADCP of targeted antibodies. Other phagocytosis checkpoints, such as CD24, PD-L1, MHC-I, STC-1, and CD22, have been identified in recent years. Antibodies targeting these checkpoints are currently undergoing preclinical studies or clinical trials (See Table 3).

While targeting phagocytosis checkpoints has introduced a new era in immunotherapy, it also faces significant challenges. First, phagocytosis checkpoints primarily control innate immune responses, which are less specific and may damage normal tissues alongside tumor cells, particularly when combined with other immune-modulating therapies, such as cytokines or STING agonists [80]. Second, the high expression of CD47 on circulating blood cells has made hematotoxicity the most prevalent side effect of CD47 inhibitors. Various strategies have been developed in the design of inhibitors to alleviate this toxicity [80]. It is believed that targeting phagocytosis checkpoints should work synergistically with T-cell responses, such as those targeting PD-L1, to enhance antitumor effects. More than half of the ongoing clinical trials are investigating combination therapies targeting CD47 (See Table 3). Collectively, further studies on the mechanisms of tumor-induced immune evasion are needed to overcome these obstacles and accelerate the development of the first drugs targeting phagocytosis checkpoints.

## Novel strategies for enhancing ADCP

Given the recognized potential of ADCP as a cytotoxic mechanism for mAbs, it is evident that novel strategies are needed to enhance and optimize the efficacy of these therapeutic regimens for cancer. Several approaches are currently being investigated to improve the efficacy of ADCP.

### Enhancing phagocytosis with nanoparticles

Extensive studies on cancer immunotherapy have shown that ADCP alone may not always be sufficient to elicit a robust anti-tumor response [81]. The use of nanoparticles may enhance phagocytic activity. Various nanoparticles, such as silica, carbon, iron oxide, or gold, have been extensively explored for their potential to promote macrophage polarization [82]. Upon recognizing these nanoparticles as foreign objects, TAMs engulf them through ADCP and subsequently release their contents in the intracellular space [83]. Consequently, nanoparticles can be loaded with therapeutic agents or substances designed to promote macrophage polarization toward a more phagocytic phenotype, effectively reprogramming them with a robust affinity for phagocytosis. This makes nanoparticles a promising vehicle for targeted delivery [84, 85].

So far, there are more than 200 ongoing clinical trials worldwide, exploring the application of various nanoparticles in the treatment of different cancers, predominantly solid tumors [86]. FDA has already approved several nanoparticles for patient use [87]. However, the potential of nanoparticles for delivering drugs to specific macrophage phenotypes remains less explored [88]. A recent study developed nanoparticles designed to selectively target M2 TAMs by delivering nanoparticles containing mRNAs for interferon regulatory factor 5 (IRF5), M1-polarizing transcription factors, and its activating kinase, IKKB [89]. The co-delivery of IRF5 and IKKB successfully induced a shift in M2 TAMs toward a pro-inflammatory, M1-like phenotype [89]. A distinctive feature of this approach is that the co-delivery does not provoke an immune response or cause systemic toxicity. Furthermore, the novelty of this study lies in the design of the targeted mRNA delivery system, which selectively interacts with mannose receptors on M2 macrophages. In vivo testing revealed that treatment with IRF5/IKKB-loaded nanoparticles significantly inhibited the progression of ovarian cancer, facilitated tumor clearance, and extended the lifespan of mice. After an intraperitoneal (i.p.) dose of 100 µg mRNA/mouse/week for 9 weeks, the ovarian cancer regressed and was eventually cleared in 40% of animals in the IRF5/IKKβ NP group (overall 142 d median survival versus 60 d in controls) [89].

This finding was further corroborated by a subsequent study in which researchers encapsulated IMD-0354, a TAM repolarization agent, in mannose-modified cationic lipid-based nanoparticles (M-IMD-CLN), and sorafenib, a kinase inhibitor for cancer treatment, in cationic lipid-based nanoparticles (SF-CLN) [90]. Mannose is selectively absorbed by M2 TAMs, which exhibit a high expression level of mannose receptors. The mannose on the nanoparticles serves as a ligand for M2 macrophage receptors, enhancing their active uptake by M2 TAMs [91]. In vivo experiments demonstrated that nanoparticles exhibited a superior biodistribution profile and can effectively localize to the tumor site, offering potent anti-tumor activity in Hepa1-6 tumor-bearing mice [90]. Furthermore, the combination of M-IMD-CLN and SF-CLN exhibited a synergistic increase in anti-tumor efficacy and TAM repolarization, compared to SF-CLN alone [90].

### CAR-macrophages

Until recently, there has been a limited number of studies on the use of CAR-engineered macrophages (CAR-Ms) [92]. A recent study by Morrissey and colleagues indicated that CAR-Ms can enhance phagocytosis. By incorporating an extracellular antibody variable fragment (ScFv) targeting CD19 and the transmembrane CD8 found in CD19 CAR-T constructs, the researchers successfully generated chimeric antigen receptors for phagocytosis (CAR-Ps) on macrophage cell line J774A.1. These CAR-Ps effectively induced the phagocytosis of antigen-coated decoy particles in an ex vivo setting [93]. The study measured the specificity of phagocytosis, guided solely by the antigen recognition ability of the ScFv domain within the CAR construct. Researchers also compared the efficacy of various intracellular domains in driving phagocytosis using a relatively simple ex vivo assay [93].

The efficacy of CAR-Ms was also supported by another study in which murine CAR-Ms were designed to specifically target the solid tumor antigen HER2. This study utilized the intracellular signaling domain of CD147 to stimulate the expression of matrix metalloproteinases (MMPs) in a HER2-dependent manner. The researchers initially stimulated HER2 expression in 4T1 cancer cells and subsequently transduced the Raw264.7 cell line with HER2-147-CAR [94]. The expression of MMP3 and MMP13 was induced upon CAR-antigen binding, leading to TME remodeling, increased T cell infiltration, and inhibition of tumor progression in vivo [94].

Recently, Carisma’s first clinical candidate, the anti-HER2 CAR-Macrophage CT-0508, was successfully validated for safety in Phase 1 (NCT04660929) clinical trial. The results demonstrated that CT-0508 was feasible to manufacture and exhibited acceptable safety and tolerability [11, 95, 96]. However, the therapeutic effects were transient, with most patients experiencing disease progression within a few months. Limited persistence of CAR-Macrophages in circulation, coupled with low infiltration levels within tumor tissues (as assessed by RNAScope in situ RNA technology), likely contributed to these outcomes. This raises critical questions regarding strategies to enhance CAR-Macrophage retention, tumor homing, and intratumoral survival [11, 94, 95]. Potential future approaches may involve genetic modifications to extend cellular lifespan, co-expression of chemokines or homing receptors, and the integration of depot-based delivery systems or repeated dosing regimens.

Interestingly, transduction with the Ad5/F35 adenovirus induced the polarization of human macrophages to a pro-inflammatory M1 phenotype independent of CAR expression [97]. Moreover, the recognition of tumor cells by CAR-Ms transformed the TME from an anti-inflammatory state to a pro-inflammatory state and activated inflammatory pathways [95]. Collectively, these findings suggest that CAR-Ms not only facilitate tumor cell phagocytosis but also alter the TME to enhance anti-tumor T cell responses [98]. A Phase 1 preclinical trial (NCT04660929) is currently underway to investigate the safety and early-stage efficacy of HER-2 CAR-Ms.

Together, leveraging CAR-Ms as an immunotherapeutic approach has facilitated the development of novel treatment options for cancer. CAR-Ms operate not only through direct phagocytosis of tumor cells but also via T cell co-stimulation and cross-presentation of vaccinal antigens [95]. Macrophages reside in tissues, and certain types of tissue-resident macrophages can survive quiescently for extended periods until activation [99–101]. This unique characteristic makes CAR-Ms ideal local sentinels for metastatic tumor cells, effectively preventing early-stage tumor relapse. Importantly, macrophage plasticity offers potential applications beyond oncology, offering new opportunities for conditions where inflammation is dispensable [102]. By designing appropriate CAR constructs, macrophages can be transformed into immunoquiescent CAR-Ms with retained phagocytic capabilities, which can help treat a range of medical conditions, including infections, neurodegenerative diseases, and autoimmune disorders. Given their broad potential, CAR-Ms have the capacity to revolutionize cancer treatment and serve as a next-generation treatment for various medical conditions [103].

### Combinatorial approaches for enhancing ADCP

ADCP can be enhanced by innate immune checkpoint inhibitors, such as CD47 blocking. Early trials have indicated that combining rituximab with an IgG4 CD47-blocking antibody can improve the response of lymphoma. The clearance of chronic lymphocytic leukemia (CLL) cells by CD20 mAbs can be enhanced by administering rituximab at low doses and high frequencies, in amounts that are adequate to stimulate ADCP without causing prolonged suppression of innate immune cytotoxicity or a significant decrease in the expression of B cell CD20. However, the full potential of ADCP as a key cytotoxic mechanism enhanced by mAb is only just beginning to be understood and exploited. Moving forward, an emphasis on combination strategies that prevent ADCP exhaustion and inhibition and promote more effective phagocytosis is expected to yield novel therapies and approaches to improve cell clearance.

## Conclusion and future perspectives

The role of macrophage phagocytosis and the subsequent cross-priming of adaptive immunity has gained significant attention in recent years. As a critical effector, it enhances adaptive anti-tumor responses. ADCP has been extensively investigated in clinical studies for more than two decades. For instance, rituximab, as the first mAb, was approved by the FDA in 1997 [11]. Since then, a wide array of innovative antibodies with diverse designs have been added to this list, many of which are either FDA-approved or currently undergoing clinical trials. Initially, this field focused on ADCP induced by therapeutic antibodies. Subsequently, it encompassed the manipulation of phagocytic checkpoints to improve therapeutic outcomes [11]. Key areas of research now include: (1) the use of immune checkpoints to enhance ADCP; (2) the use of nanoparticles to enhance the phagocytic capacity of macrophages; (3) the incorporation of CAR constructs into macrophages to increase the specificity of targeted antigens; (4) combinatorial approaches for enhancing ADCP; and (5) investigating novel intravital imaging for the real-time monitoring of ADCP events. These groundbreaking advancements have significantly propelled macrophage-mediated phagocytosis of cancer, making it a promising avenue for cell-based treatment [11].

The CD47–SIRPa axis was the first phagocytosis checkpoint identified in cancer, and subsequent discoveries have revealed additional phagocytosis checkpoints, including PD-1/PD-L1 and CD24–Siglec-10 [53, 63, 104, 105]. Phagocytosis checkpoint blockade therapies have shown promising results in clinical trials, exemplified by CD47-blocking therapeutics such as Magrolimab in non-Hodgkin lymphoma and acute myeloid leukemia, as well as TTI-622 in lymphoma [11]. Due to the distinct yet complementary immunological pathways activated by phagocytosis checkpoint blockade, macrophage phagocytosis emerges as a promising therapeutic strategy, comparable to established treatments like PD-1 inhibitors [106]. However, the effectiveness of phagocytosis checkpoint blockades, particularly those in the form of monoclonal or bispecific antibodies for targeting metastatic tumors, remains to be thoroughly investigated.

The prospect of CAR-M-based immunotherapy has been significantly advanced by earlier findings from Zhang et al. and Morrissey et al. [93, 94]. Klichinsky et al. pioneered the use of macrophages with a CAR construct and harnessed the power of macrophage-mediated phagocytosis alongside antigen specificity to boost anti-cancer effects, yielding results comparable to CAR-T cell therapies [95]. In addition to overcoming the challenges of transducing primary macrophages, they took advantage of macrophage plasticity to induce an M1-like phenotype, simultaneously remodeling the TME into a more pro-inflammatory state. These promising findings are now undergoing clinical trials, starting with a CAR-M targeting HER2. While CAR-Ms show great promise as a novel immuno-oncology approach, further development of a robust pipeline is required to fully assess their efficacy and safety profile. For instance, as with CAR-T therapies, cytokine release syndrome (CRS) remains a significant challenge. CRS is primarily mediated by the activation of macrophages, leading to the rapid and excessive release of pro-inflammatory cytokines [107]. Therefore, the safety profile of CAR-Ms warrants further investigation. Another critical consideration is the design of CAR constructs, with an emphasis on optimizing phagocytosis signaling, enhancing specificity, and minimizing potential toxicity. Overall, CAR-M-based immunotherapy represents an emerging and highly promising direction for future research in this field.

In terms of clinical relevance, engineered macrophages hold promise not only as monotherapies but also as complementary therapeutics in multimodal treatment regimens. For example, combining CAR-Ms with anti-PD-1 or anti-CTLA-4 may enhance cytotoxicity and overcome T cell exhaustion in immunologically “cold” melanoma [108]. Moreover, early-phase clinical trials of engineered macrophages in a variety of malignancies (e.g., glioblastoma) provide valuable safety data that could inform the development of melanoma-specific therapeutic protocols [108]. These examples highlight the translational potential of macrophage-based platforms in both standalone and combination therapeutic approaches.

Several clinical trials are currently underway to evaluate therapies specifically targeting TAMs via nanoparticles. These trials, along with other original research studies, have generated promising results that may soon be applicable to patient care [109]. However, significant work remains to establish the feasibility of TAM targeting through nanomedicine for optimal clinical translation. For example, a key question that remains unanswered is how to optimize the trafficking and tumor localization of nanoparticles. Despite leveraging the enhanced permeability and retention effect to promote nanoparticle accumulation through leaky tumor vasculature, studies have shown that only approximately 1% of nanoparticles successfully reach the tumor site [110]. This represents a critical barrier that must be overcome before expanding the applications of nanomedicine. Furthermore, another challenge is ensuring the persistence of nanoparticles in vivo and preventing their rapid clearance by the immune system. Even if nanoparticles can overcome the challenges of trafficking and tumor localization, the immune system may still identify them as foreign entities and eliminate them prior to exerting their therapeutic effects. To address this, a potential strategy could involve applying the principle outlined by Deuse et al., which involves generating hypoimmunogenic nanoparticles that evade immune elimination. This approach could enhance nanoparticle persistence and facilitate drug delivery by suppressing major histocompatibility complex (MHC) I and II expression while overexpressing the CD47 molecule [111]. Given that these factors are significant obstacles to the efficacy of nanomedicine in inducing potent M1 TAM activity, substantial further research is needed to optimize this promising and innovative form of immunotherapy.

Looking ahead, future research should focus on the investigation of multi-target CAR-Ms to antigenic heterogeneity. In addition, the integration of High-resolution omics technologies, such as spatial proteomics and single-cell transcriptomics, may provide deeper insights into macrophage-TME crosstalk, thus facilitating the rational design of therapeutic strategies [108]. Furthermore, the integration of machine learning and artificial intelligence (AI) could aid in identifying optimal macrophage engineering approaches tailored to specific molecular signatures [108].

Ultimately, intravital imaging, a crucial component in the preclinical design of immunotherapies, should be integrated into novel approaches to optimize cancer diagnosis and immunotherapies. For instance, this imaging technique can be coupled with other modalities, such as dynamic in situ cytometry (DISC), which utilizes cell surface labeling to correlate phenotypic markers with motility parameters [44]. In addition, intravital imaging can be paired with radiopharmaceuticals and antibody-based contrast agents to facilitate the robust monitoring of ADCP. Notably, intravital imaging can be integrated with advanced methods, including Clustered Regularly Interspaced Short Palindromic Repeats (CRISPR/Cas9), mass spectrometry, spatial transcriptomics, Labeling Immune Partnership by Sorting Intercellular Contacts (LIPSTIC), and single-cell sequencing [44]. These integrations will further solidify intravital imaging as an indispensable and unique tool, deepening our understanding of immune cell dynamics and behavior within the TME and guiding immunotherapeutic interventions for cancers.

## Data Availability

Data will be made available on request.

## Abbreviations

FDA: Food and Drug Administration
ADCP: Antibody-dependent cell phagocytosis
mAbs: monoclonal antibodies
ICIs: Immune checkpoint inhibitors
CAR: Chimeric antigen receptor
TAMs: Tumor-associated macrophages
TME: Tumor Microenvironment
PRRs: Pattern recognition receptors
FcgRs: Fc gamma receptors
Fc: Fragment crystallizable
ITAMs: Immunoreceptor tyrosine-based activation motifs
ITIM: Immunoreceptor tyrosine-based inhibition motif
NHL: Non-Hodgkin lymphoma
CD20: Cluster of Differentiation 20
SIRPα: Signal Regulatory Protein Alpha
SLAMF7: Signaling Lymphocytic Activation Molecule Family Member 7
SCID: Severe combined immunodeficiency
L540cy: Disseminated Hodgkin disease model
PD-L1: Programmed Cell Death Ligand 1
DCs: Dendritic cells
CNS: Central nervous system
NSCLC: Non-small cell lung cancer
ROS: Reactive oxygen species
SSG: Sodium stibogluconate
AML: Acute myeloid leukemia
AZA: Azathioprine
HCC: Hepatocellular carcinoma
SCLC: Small cell lung cancer
TNBC: Triple-Negative Breast Cancer
ICAM-3: Intracellular adhesion molecule 3
MAC-1: Macrophage-1 antigen
CR3: Complement receptor 3
Src: Src family tyrosine kinase
Syk: Spleen tyrosine kinase
Btk: Bruton’s tyrosine kinase
IgG: Immunoglobulin G
TP53: Tumor Protein p53
PD-1: Programmed Cell Death Protein 1
NSG: NOD SCID gamma
LPS: Lipopolysaccharide
TLR4: Toll-Like Receptor 4
STAT6: Signal Transducer and Activator of Transcription 6
mTOR: mechanistic target of rapamycin
MHC-II: Major Histocompatibility Complex class II
TNFα: Tumor necrosis factor-α
IL-12: Interleukin-12
PFS: Progression-free survival
CAR-T: Chimeric Antigen Receptor T-Cell Immunotherapy
KCs: Kupffer Cells
EGFR: Epidermal Growth Factor Receptor
STC-1: Stanniocalcin-1
M-IMD-CLN: mannose-modified cationic lipid-based nanoparticles
SF-CLN: cationic lipid-based nanoparticles
CAR-Ms: CAR-engineered macrophages
ScFv: Extracellular antibody variable fragment
CAR-Ps: Chimeric antigen receptors for phagocytosis
HER2: Human Epidermal Growth Factor Receptor 2
MMPs: Matrix metalloproteinases
CLL: Chronic lymphocytic leukemia
